# Subjective visual sensitivity in neurotypical adults: insights from a magnetic resonance spectroscopy study

**DOI:** 10.3389/fnins.2024.1417996

**Published:** 2024-09-25

**Authors:** Lenka Jurkovičová, Julie Páleník, Petr Kudlička, Lenka Pezlar, Alexandra Ružičková, Vojtěch Juřík, Radek Mareček, Robert Roman, Jason J. Braithwaite, Kristian Sandberg, Jamie Near, Milan Brázdil

**Affiliations:** ^1^First Department of Neurology, St. Anne’s University Hospital and Medical Faculty of Masaryk University, Brno, Czechia; ^2^CEITEC–Central European Institute of Technology, Masaryk University, Brno, Czechia; ^3^Department of Psychology, Faculty of Arts, Masaryk University, Brno, Czechia; ^4^Institute of Computer Aided Engineering and Computer Science, Faculty of Civil Engineering, Brno University of Technology, Brno, Czechia; ^5^Department of Psychology, Lancaster University, Lancaster, United Kingdom; ^6^Center of Functionally Integrative Neuroscience, Aarhus University, Aarhus, Denmark; ^7^Physical Sciences, Sunnybrook Research Institute, Toronto, ON, Canada

**Keywords:** Pattern Glare Test, visual discomfort, magnetic resonance spectroscopy, GABA, glutamate, cortical excitability

## Abstract

**Introduction:**

Altered subjective visual sensitivity manifests as feelings of discomfort or overload elicited by intense and irritative visual stimuli. This can result in a host of visual aberrations including visual distortions, elementary visual hallucinations and visceral responses like dizziness and nausea, collectively referred to as “pattern glare.” Current knowledge of the underlying neural mechanisms has focused on overall excitability of the visual cortex, but the individual contribution of excitatory and inhibitory systems has not yet been quantified.

**Methods:**

In this study, we focus on the role of glutamate and γ-aminobutyric acid (GABA) as potential mediators of individual differences in subjective visual sensitivity, measured by a computerized Pattern Glare Test—a series of monochromatic square-wave gratings with three different spatial frequencies, while controlling for psychological variables related to sensory sensitivity with multiple questionnaires. Resting neurotransmitter concentrations in primary visual cortex (V1) and right anterior insula were studied in 160 healthy participants using magnetic resonance spectroscopy.

**Results:**

Data showed significant differences in the perception of visual distortions (VD) and comfort scores between men and women, with women generally reporting more VD, and therefore the modulatory effect of sex was considered in a further examination. A general linear model analysis showed a negative effect of occipital glutamate on a number of reported visual distortions, but also a significant role of several background psychological traits. When assessing comfort scores in women, an important intervening variable was the menstrual cycle.

**Discussion:**

Our findings do not support that baseline neurotransmitter levels have a significant role in overreactivity to aversive stimuli in neurotypical population. However, we demonstrated that biological sex can have a significant impact on subjective responses. Based on this additional finding, we suggest that future studies investigate aversive visual stimuli while examining the role of biological sex.

## 1 Introduction

Certain individuals are more sensitive to harsh lights or patterns than others – resulting in the experience of visual discomfort, sensory overload, irritation, and anxiety or anger. This subjective feeling is called subjective visual sensitivity and varies across individuals both within the neurotypical population and in association with certain disorders like autism or migraine (Braithwaite et al., 2013; Robertson and Simmons, 2013; Braithwaite et al., 2015; Ward, 2019; Wood et al., 2021). Proposed neural mechanisms for inter-individual differences involve a change in the balance of excitatory and inhibitory systems, but direct evidence quantifying the individual contribution of these systems is lacking.

Individual differences in subjective sensory sensitivity can be studied with laboratory tasks that utilize aversive stimuli, such as the Pattern Glare Test (PGT, Wilkins et al., 1984; Evans and Stevenson, 2008; Braithwaite et al., 2013), aiming at assessing particularly visual sensitivity and resultant visual distortions/aberrations. This visual task features stationary high-contrast horizontal achromatic gratings with different spatial frequencies that can elicit discomfort, induce phantom visual perceptions, and visual distortions (e.g., colorful halos, shadows and illusory movement) as well as visceral responses like nausea and dizziness. These experiences are a form of “visual stress,” collectively referred to as *pattern glare*. Gratings with a spatial frequency of around 3 cycles-per-degree (cpd) are particularly potent at inducing pattern glare in observers (Wilkins et al., 1984; Braithwaite et al., 2013) and even more so in hypersensitive persons, e.g., those suffering from migraine (Huang et al., 2003; Fong et al., 2019; Fong et al., 2020). Multiple neural mechanisms for this effect have been proposed, ranging from pre-cortical mechanisms as early as at the retina (Szmajda and Devries, 2011) to post-sensory centrally mediated processing including cognitive-affective responses (Green and Wood, 2019).

Increased excitability of V1 has been considered a plausible mechanism of subjective visual sensitivity since early theories (Wilkins et al., 1984), supported by later research in migraine patients (Wilkins et al., 2004). Spatial frequencies around 3 cpd are rare in natural scenes (Conlon et al., 2001; Geisler, 2008; Haigh et al., 2015), therefore V1 is not efficient in their encoding and responds with unnecessarily abundant activation (De Valois et al., 1974; Le et al., 2017). This makes these frequencies more likely to overstimulate the visual cortex; for example to trigger epileptic seizures (Radhakrishnan et al., 2005). This overall increase in neural activation (a kind of over-stimulation) might reflect increased excitation, decreased inhibition, or both. Excitation is primarily facilitated by glutamate and inhibition by γ-aminobutyric acid (GABA) (Badawy et al., 2012). The basic processing microcircuit in the cerebral cortex consists of excitatory glutamatergic projection neurons and inhibitory GABAergic interneurons (Douglas and Martin, 2004). In case of intense stimulation of a single type of frequency-sensitive cells, the excitation might exceed the shared lateral inhibitory capacity of the microcircuit (Evans and Stevenson, 2008). Therefore, uncomfortable striped patterns overstimulate the neurons and produce larger and less sparse activation in a computational model (Hibbard and O’Hare, 2015), resembling the excessive activation of the brain during sensory overload. As this occurs in the visual cortex, these processes manifest themselves as increased susceptibility to visual pattern glare experiences.

Direct evidence for the role of visual cortex excitability in subjective visual sensitivity comes from neuroimaging research. In functional magnetic resonance imaging (fMRI) studies, uncomfortable striped patterns evoke increased blood oxygenation response in V1 and visual association cortex (Huang et al., 2003; Huang et al., 2011). This has been corroborated by near infrared spectroscopy (Haigh et al., 2013), and electrophysiology (Adjamian et al., 2004; O’Hare et al., 2015; O’Hare, 2017; Orekhova et al., 2019). Causal evidence for the role of cortical excitability comes from transcranial direct current stimulation (Braithwaite et al., 2015), where under excitatory (anodal) stimulation of V1, healthy subjects perceived more visual distortions on medium-frequency gratings and this effect was larger for observers screened for trait-based predisposition to anomalous perceptions. Although these findings point to the role of increased excitation-to-inhibition ratio in subjective visual sensitivity, the individual role of excitatory glutamatergic and inhibitory GABAergic systems awaits clarification. Currently, the only non-invasive method measuring GABA and glutamate concentrations *in vivo* is proton magnetic resonance spectroscopy (MRS) (Öz et al., 2020). MRS-quantified GABA and glutamate concentrations have been previously found to reflect change in the level of cortical excitability as measured (Stagg et al., 2011a) or manipulated (Gröhn et al., 2019) by transcranial magnetic stimulation and also to reflect the role of GABA in visual perception (Song et al., 2017).

Additional evidence on the role of cortical excitability, not limited only to V1, arises from studies in migraine patients where patients proved to be particularly susceptible to pattern glare (Wilkins and Evans, 2010; Fong et al., 2020). Patients suffering from so-called complex auras show higher resting-state functional connectivity within the visual network and the right anterior insula (rAI) (Silvestro et al., 2022), which also shows heightened inter-ictal intrinsic connectivity with V1 in migraine without aura (Tso et al., 2015). The anterior insula, as a key node of the salience network, evaluates the impact of sensory stimuli on the body state (Downar et al., 2000; Cauda et al., 2011; Uddin, 2015) and along with the visual and parietal brain areas, is involved in multisensory and cognitive-affective processing—including the generation of conscious feeling states (Saffin and Tohid, 2016; Gogolla, 2017; Campbell et al., 2018; Cebeiro and Rodríguez, 2019). The rAI cortex has a role in bodily awareness and interoception (Craig, 2009; Rahmani and Rahmani, 2019; Fermin et al., 2021). Consequently, the insula may well be important for mediating the visceral-body related experiences reported from viewing aversive gratings.

In the present study, we aim to expand the understanding about the role of cortical excitability in subjective visual sensitivity by quantifying the contribution of baseline GABA and glutamate, utilizing naturally occurring inter-individual differences in a neurotypical sample. To measure visual sensitivity, we used both measures of the PGT: aberrant visual experiences (visual distortions—VD) and subjective ratings of visual discomfort. We related these scales to glutamate and GABA concentrations measured with proton magnetic resonance spectroscopy in V1 and in the rAI, while controlling for response bias and predisposition towards anomalous experiences with multiple questionnaires. We predicted that: (1) the number of visual distortions elicited by aversive medium-frequency gratings would be negatively correlated to inhibitory GABA or (2) positively correlated to excitatory glutamate in V1; (3) subjectively reported feeling of comfort would be positively correlated to GABA or (4) negatively correlated to glutamate in V1. We aimed to also evaluate the role of rAI excitability in a context of subjective visual sensitivity and propose a model of the relationship between cortical excitability and subjective sensitivity. By applying the hyperexcitability hypothesis on young neurotypical adults, we attempt to bridge the explanatory gap between aberrant neural processes and anomalous conscious perceptions in neurotypical samples.

## 2 Materials and methods

One hundred eighty five healthy young adults (aged 18 to 39; mean = 24.28, SD = 4.762) with normal or corrected-to-normal vision and no neurological or psychiatric diagnosis were recruited via a database of volunteers and advertisements in university/social media. The volunteers were invited to participate in the research as a part of an international research project on consciousness research (COST Action CA18106–the neural architecture of consciousness), for which the exclusion criteria were adapted. With respect to these criteria, we excluded individuals over 40 years of age, with current neurological or psychiatric medication intake, a history of migraine symptoms with aura or those not fulfilling MR safety criteria, as they self-reported in a screening questionnaire prior to study participation. In total, 182 subjects (self-reported 72 males and 110 females) gave written informed consent approved by the Research Ethics Committee of Masaryk University and underwent both the PGT and magnetic resonance spectroscopy. Participants were asked not to drink caffeinated beverages for at least 4 h before the first session (Wolde, 2014). After completing experiments, the subjects were debriefed and received a financial compensation of 1,000 Czech crowns (~40 EUR).

### 2.1 Questionnaires

Validated psychological questionnaire measures were administered to provide an index of participants’ trait-based predispositions to anomalous perceptions and subjective sensitivity that might influence the perception of visually aversive patterns. The questionnaires were selected to ascertain individual scores on various psychological aspects related to sensory sensitivity and with regard to the previous research on the topic (Braithwaite et al., 2013; Dance et al., 2021). This was supplemented by demography, sleep, and menstrual cycle.

#### 2.1.1 Cardiff Anomalous Perceptions Scale

*Cardiff Anomalous Perceptions Scale (CAPS)* (Bell et al., 2006) is an instrument for measuring the propensity to report anomalous perceptual experiences, hallucinations in non-clinical populations. The questionnaire consists of 32 items of different forms (open-closed questions and Likert scales), divided into 3 components that can be interpreted as “clinical psychosis,” “chemosensation,” and “temporal lobe disturbance.” Besides a total score that can be calculated by summing the number of endorsed items, it produces three separate subscale scores measuring distress, intrusiveness and frequency. Therefore, the possible range for the CAPS total was 0 (low) to 32 (high), and for each of the dimensions the possible range is 0 to 160.

#### 2.1.2 Glasgow Sensory Questionnaire

*Glasgow Sensory Questionnaire (GSQ)* (Robertson and Simmons, 2013) assesses self-rated hyper- and hypo-sensitivities across seven sensory modalities: visual, auditory, tactile, gustatory, olfactory, proprioceptive, and vestibular. The questionnaire consists of 42 items, six items targeting each sensory domain. Half of these items measure hypersensitivity, while the other half examine hyposensitivity. Each item can be answered using a scale of 0 (“Never”), 1 (“Rarely”), 2 (“Sometimes”), 3 (“Often”), and 4 (“Always”), the overall sensitivity score is calculated by summing all item scores (ranging 0 to 168). From the overall score, two separate scores can be derived for hyper-and hyposensitivity (ranging from 0 to 84), as well as one score for every sensory domain (ranging 0 to 24).

#### 2.1.3 NEO-FFI

*NEO Five-Factor Inventory* (NEO-FFI; Costa, 1989; Costa and McCrae, 1992) is a revised, short version of NEO Personality Inventory (Costa and McCrae, 1985). It consists of 60 items providing a concise measure of five personality factors: neuroticism, extraversion, openness, agreeableness, conscientiousness. Each of the factors is loaded with 12 items, some of which (N = 28) are reverse-worded. The questionnaire uses a five-point Likert response format to indicate if participants (0) strongly agree, (1) agree, (2) are neutral, (3) disagree or (4) strongly disagree with a given proposition about themselves. Scores for each personality factor are calculated by summing 12 items with reverse-scored reversed items.

#### 2.1.4 Multidimensional Assessment of Interoceptive Awareness-2

*Multidimensional Assessment of Interoceptive Awareness-2 (MAIA-2)* (Mehling et al., 2012) was designed as a multidimensional self-report measure to assess the main psychological aspects of the perception of body sensations. The 37 included items may be divided into eight subscales providing separate scores. The subscales are: noticing, trusting, body listening, emotional awareness, self-regulation, not worrying, not distracting and attention regulation. Participants assign 6-point Likert ratings from 0 (“Never”) to 5 (“Always”). Lower sum of scores (an overall or for the certain subscale) indicate more deficits in interoceptive awareness.

#### 2.1.5 Biological factors

Studies have attempted to provide normative data for cortical excitability parameters, from which circadian regulation and menstrual/ovarian cycle serve as potential bias factors in our study. Sleep deprivation increases cortical excitability significantly (Meisel et al., 2015). There is some evidence that there are no differences resulting from sex (Cueva et al., 2016), but studies focused on menstrual and ovarian cycle in women proved the effect of ovarian hormones to be an important factor affecting cortical excitability since menstrual cycle causes the fluctuations in the neurotransmitter concentrations (Smith et al., 1999; Smith et al., 2002; Inghilleri et al., 2004; Hattemer et al., 2007). Therefore, we decided to gather participants’ data on the hours slept during the night before the experiment and the day of the menstrual cycle in women. A normal menstrual cycle is defined here as a standard 21–35 day cycle that is not regulated by hormonal contraceptives.

### 2.2 Pattern Glare Test

**Figure 1:**
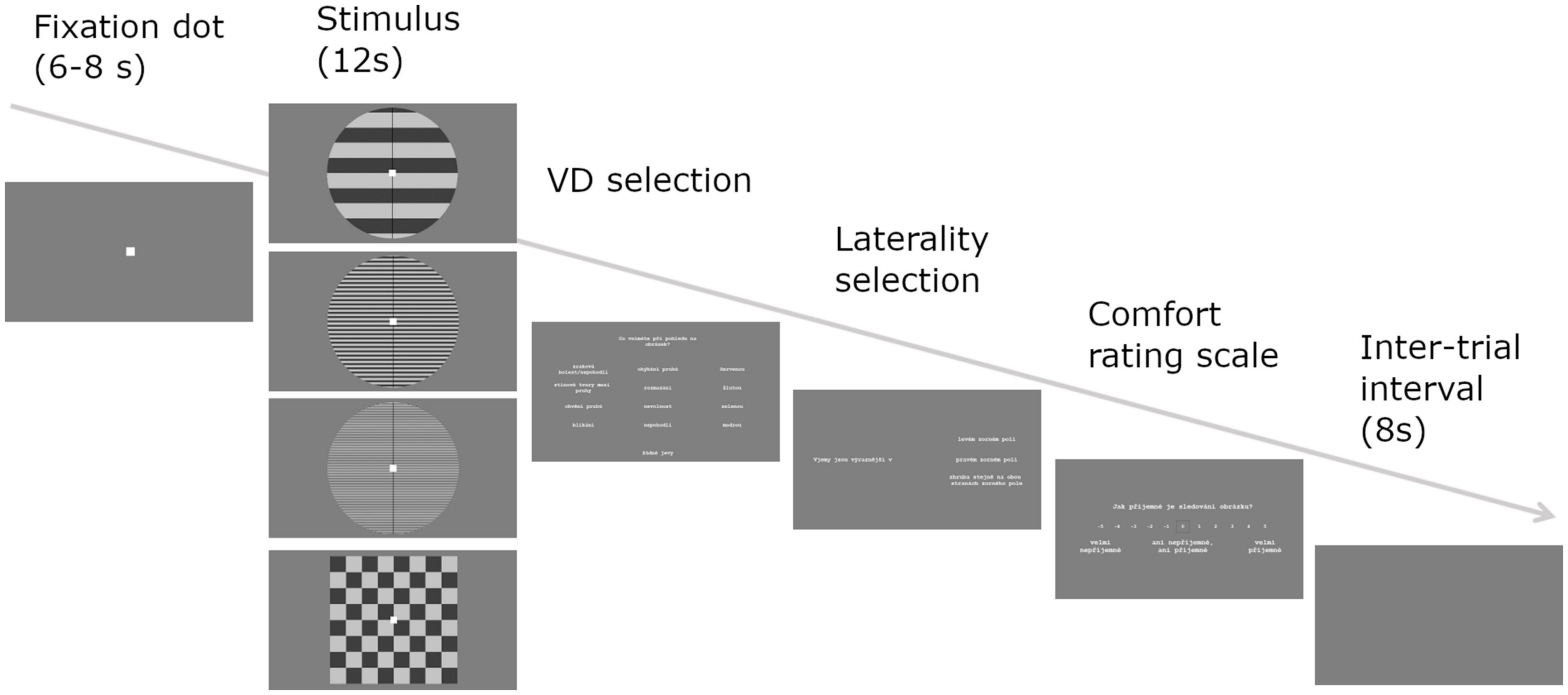
Pattern Glare Test trial. Fixation dot was followed by one of four stimuli and all three response screens: VD selection—What did you perceive when looking at the image? (shadowy shapes amongst the lines, shimmering, flickering, bending, illusory stripes, red, blue, yellow, green, nausea, dizziness, ocular pain; select as many as perceived). Laterality selection—the distortions were more prominent on: left side, both sides about the same, right side (select). Comfort rating—How comfortable was looking at the image? Rate on a scale: −5 = very uncomfortable, 0 = neither comfortable nor uncomfortable, 5 = very comfortable.

With the aim to assess state-based subjective visual sensitivity, we used a modified computerized version of the Pattern Glare Test (Braithwaite et al., 2015), see Figure 1. The stimulation consisted of stationary horizontal square-wave achromatic gratings differing only in their spatial frequency. Three frequencies were presented: a control low-frequency grating (0.5 cpd – cycles pre degree) intended to screen for response bias, an aversive medium-frequency grating (3 cpd), and high-frequency grating (14 cpd). Each grating was presented 6 times in a randomized order. After every three trials with grating stimuli, a checkerboard of 0.5 cpd was presented instead to reduce the potential for lingering excessive neural activity to carry over onto subsequent stimuli. The task was administered in a shielded laboratory and the subject was seated at 80 cm distance from the presentation monitor (TFT-LCD display Philips 241S4L, refresh rate 60 Hz, 1920 × 1,080 pixels, 533 × 300 mm). The gratings had dimensions 120 × 120 mm (432 × 432 pixels). The Michelson contrast of the gratings was 0.7, the background luminance 50 lux. The light in the room was kept on a stable dim setting (35 lux).

At the beginning of the experiment, participants were instructed on the definitions of visual distortions, and then to sit comfortably with one hand on the mouse and the other on the keyboard. Their basic task was to fixate a point at the center of the screen for the whole duration of its presence. If a stimulus turned out too uncomfortable, the participant could remove it by pressing the spacebar. Another spacebar press restored the stimulus. After each stimulus, participants were presented with three response screens: 1) select from a list all perceived visual distortions, 2) mark prevailing laterality of visual distortions (left/center/right) and 3) rate their comfort with viewing the stimulus on a 11-point scale (Figure 1). At the start of the experiment, two practice trials with checkerboard stimulus were included to ensure that participants understood the task. At the end, participants were debriefed and asked to report any visual distortions that did not fit the available alternatives. The whole task took approximately 15 min.

The number of visual distortions for each frequency (low-frequency gratings—VD-low, medium-frequency gratings—VD-med, high-frequency gratings—VD-high) was calculated as the average of the number of distortions reported after each presentation. We took into account the distortions of visual nature, i.e. shadowy shapes amongst the lines, shimmering, flickering, bending, illusory stripes or colors, and overall discomfort such as nausea, unease, dizziness, and ocular pain. We also utilized a second measure of visual sensitivity - the comfort score, which was calculated by averaging the comfort rating from each of the six stimulus presentations for each stimulus frequency (Comfort-low, Comfort-med, Comfort-high).

Data were examined for outliers and data from 12 participants who diverted from the instructions were excluded from further analysis. Seven participants did not pass the screening on the control VD-low for response bias by reporting over 2.64 distortions at average (>2.5 SD) and five by reporting more distortions on VD-low than to the aversive grating (Evans and Stevenson, 2008).

### 2.3 MRI scan

To quantify the concentrations of individual neurotransmitters, we used the only currently available non-invasive method for measuring GABA and glutamate concentrations *in vivo*—magnetic resonance spectroscopy (MRS, Öz et al., 2020). MRS-quantified GABA and glutamate concentrations have been previously found to reflect change in the level of cortical excitability as measured (Stagg et al., 2011a) or manipulated (Gröhn et al., 2019) by transcranial magnetic stimulation and also to reflect the role of GABA in visual perception (Song et al., 2017). Participants underwent MRI scanning on the same day as PGT was performed.

**Figure 2:**
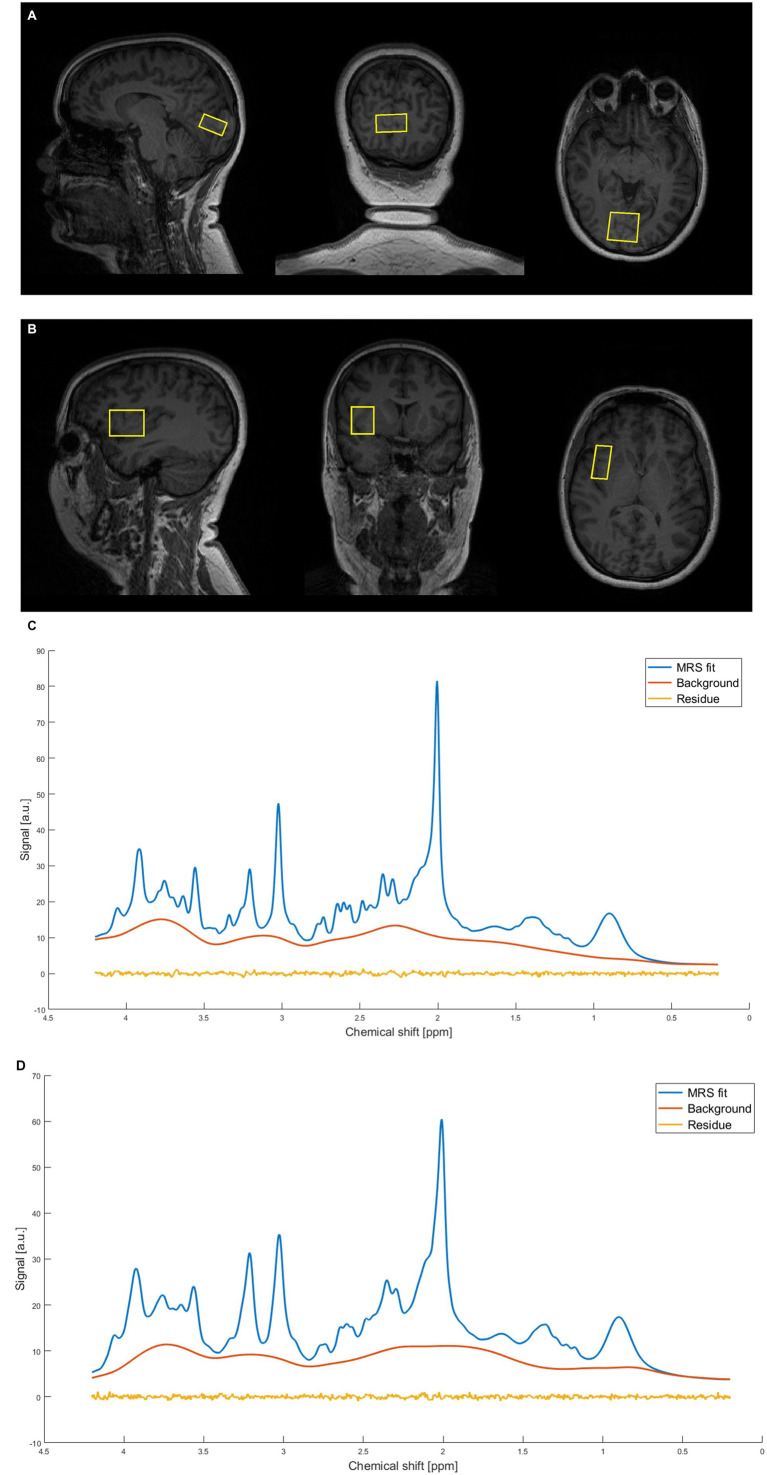
Representative example of magnetic resonance spectra from a single subject. **(A)** Occipital voxel placement. **(B)** Right anterior insula voxel placement. **(C)** Representative spectrum calculated by LCModel. The figure shows MRS fit, baseline and residuum for occipital voxel. **(D)** Representative spectrum calculated by LCModel. The figure shows MRS fit, baseline and residuum for insular voxel.

Neuroimaging data were collected in a 3 Tesla MRI Scanner (MAGNETOM Prisma, Siemens Medical, Erlangen, Germany, Syngo VE11) using a 64-channel receive-array head/neck coil. Structural T1-weighted images were acquired during each measurement using a standard magnetization-prepared rapid gradient-echo (MPRAGE) sequence (T_R_/T_E_ = 2300/2.34 ms, T_I_ = 900 ms, flip angle = 8°, slice thickness = 1 mm, 240 slices, field-of-view = 260 × 256 mm, resolution = 1 mm isotropic) for accurate placement of the MRS volume of interest (VOI) and within-VOI brain segmentation (Lin et al., 2021). MRS data were acquired using the SPECIAL sequence (Mekle et al., 2009; Near et al., 2013). The first voxel was placed in the primary visual cortex placed to cover the calcarine sulcus bilaterally. The calcarine sulcus is a prominent anatomical landmark in the T1-weighted MPRAGE structural MRI scans, and a commonly used landmark for localization of the primary visual cortex. Thus, the V1 voxel was centered on this landmark, as shown in Figure 2A. The voxel is placed as much as possible over the primary visual cortex without contaminating skull signals and includes V1 and a minimal part of the prestriate cortex. The second voxel was in the right anterior insula (Figure 2B), focused to include the whole anterior insula and as minimal part of posterior insula as possible, given the inter-individual differences in brain volume. Both voxels had these parameters: VOI = 30 × 15 × 25 mm, T_R_/T_E_ = 3000/8.5 ms, 128 NEX, AT = ~6:36 min., VAPOR water suppression with 66° flip angle (Tkác et al., 1999). Unsuppressed water spectra (8 NEX) were acquired as the internal reference for metabolite quantification in absolute and relative units and correction of residual eddy currents. GRE brain SIEMENS shimming was used for shimming the MRS sequences. The straightforward MRS-VOI positioning secured its reproducible placement by a single operator (Park et al., 2016). MRS data were obtained with participants instructed to keep their eyes closed.

The advanced SPECIAL MRS method at 3T was used as it bears several advantages over the more conventional spectral editing techniques, such as superior localization performance, low sensitivity to B1 inhomogeneities and short echo time (Öz et al., 2020). Importantly, it allows reliable quantification of several metabolites simultaneously, including both GABA and glutamate as the main neurotransmitters of interest in this study, while maintaining reproducibility comparable to previously published reproducibility values for edited GABA measurements. The SPECIAL sequence was chosen for detection of GABA for the following reasons: 1) It uses a short echo time, this minimizing decay of the GABA resonances due to T2 relaxation and scalar evolution; 2) It maximizes GABA signal by preserving all three of the GABA resonances, compared to the difference editing approaches in which typically ~ 50% of the C4 and C2 GABA resonances is removed and ~ 100% of the C3 GABA resonance is removed due to the editing process; 3) It enables simultaneous detection and quantification of a large number of other metabolites, due to the short echo time. Moreover, LCModel has been shown to reliably estimate the concentration of GABA in synthetic SPECIAL MRS data with known GABA concentrations (Near et al., 2013). However, although the SPECIAL sequence demonstrates effective removal of macromolecule contamination (Near et al., 2011), it is acknowledged that the GABA concentration estimate may still contain some signal contributions from macromolecules and other sources (e.g., homocarnosine).

#### 2.3.1 MRI/MRS data processing

MRS data were processed using the FID appliance (FID-A), an open-source MATLAB-based toolkit (Simpson et al., 2017). The FID-A processing pipeline had several steps including: combination of multiple coils, alignment of SPECIAL subspectra, removal of motion-corrupted averages, and spectral registration for correction of frequency and phase drift. Brain metabolites were quantified with LCModel (Provencher, 1993; Pfeuffer et al., 1999; Provencher, 2001; Tkác et al., 2009) using a simulated basis set containing the following metabolites: Alanine (Ala), Aspartate (Asp), Phosphocholine (PCh), Creatine (Cr), Phosphocreatine (PCr), GABA, Glutamine (Gln), Glutamate (Glu), Glutathione (GSH), Glycine (Gly), Myo-inositol (mIns), Lactate (Lac), N-acetylaspartate (NAA), Scyllo-inositol (sIns), Taurine (Tau), Glucose (Glc), N-acetylaspartylglutamate (NAAG), Glycerophosphocholine (GPC), Phosphorylethanolamine (PE), Serine (Ser), and beta-hydroxybutyrate (bHB). CSF, GM, WM fractions were calculated using GABA ANALYSIS TOOLKIT, Gannet 2.1 (Edden et al., 2014; Harris et al., 2015). Measured signal was corrected for the CSF-fraction of the voxel for 12 metabolites (Dhamala et al., 2019) including γ-aminobutyric acid (GABA) and glutamate (Glu), see descriptives in Tables 1, 2. Also a measured spectrum of fast-relaxing macromolecules (MM) was included in the basis set, based on an average metabolite-nulled brain macromolecular spectra acquired in six healthy volunteers. We excluded from the dataset 10 participants with low data quality: four in the V1 set and six in the set from insula. The inclusion criteria were signal-to-noise ratio (SNR) > = 30, water linewidth <= 0,05 ppm, and good fit of LCModel (based on visual check of fit, baseline and residuals), see Figures 2C,D. The SNR and the FWHM (full width at half maximum) were determined by the program LCModel (Provencher, 1993). SNR is defined here as the ratio of the maximum in the spectrum minus baseline over the analysis window to twice the root mean square residuals. FWHM is a rough estimate of the linewidth in the *in vivo* spectrum. The maximum peak in the spectrum is NAA. Metabolites for which a single metabolite gives an average Cramèr-Rao lower bounds (CRLB) value >20% across all participants were excluded (Kreis, 2016).

**Table 1:** Average and standard deviation of metabolite concentrations and ratios to total creatine across all participants, with separate data for males and females, alongside average CRLB values, presented for the occipital voxel.

V1	All sample, *N* = 160			Males, *N* = 65			Females, *N* = 95		
	Concentration (Mean ± SD)	Ratio to tCr (Mean ± SD)	Mean CRLB (%)	Concentration (Mean ± SD)	Ratio to tCr (Mean ± SD)	Mean CRLB (%)	Concentration (Mean ± SD)	Ratio to tCr (Mean ± SD)	Mean CRLB (%)
GABA	3.3 ± 0.55	0.3 ± 0.05	11.73	3.25 ± 0.54	0.3 ± 0.06	12.11	3.33 ± 0.56	0.31 ± 0.05	11.46
Glu	10.1 ± 0.83	0.93 ± 0.07	4.01	10.01 ± 0.77	0.92 ± 0.07	4.09	10.16 ± 0.87	0.93 ± 0.08	3.96
Gln	2.32 ± 0.45	0.21 ± 0.04	15.34	2.45 ± 0.43	0.23 ± 0.04	15.06	2.23 ± 0.46	0.21 ± 0.04	15.54
Asp	4.45 ± 0.49	0.41 ± 0.05	7.56	4.43 ± 0.42	0.41 ± 0.04	7.72	4.47 ± 0.54	0.41 ± 0.05	7.45
GSH	2.2 ± 0.19	0.2 ± 0.02	6.18	2.19 ± 0.2	0.2 ± 0.02	6.29	2.2 ± 0.18	0.2 ± 0.02	6.11
Ins	7.72 ± 0.89	0.71 ± 0.07	4.41	7.75 ± 0.89	0.71 ± 0.08	4.48	7.7 ± 0.89	0.7 ± 0.07	4.37
Lac	0.59 ± 0.25	0.05 ± 0.02	45.19	0.61 ± 0.26	0.06 ± 0.03	44.95	0.58 ± 0.24	0.05 ± 0.02	45.35
NAA	15.85 ± 0.96	1.46 ± 0.1	1.9	15.82 ± 0.94	1.46 ± 0.1	1.91	15.87 ± 0.97	1.46 ± 0.1	1.89
Scyllo	0.37 ± 0.11	0.03 ± 0.01	18.58	0.33 ± 0.1	0.03 ± 0.01	21.08	0.4 ± 0.12	0.04 ± 0.01	16.87
NAAG	1.46 ± 0.19	0.13 ± 0.02	10.83	1.45 ± 0.21	0.13 ± 0.02	11.22	1.48 ± 0.17	0.14 ± 0.02	10.56
tCh	1.33 ± 0.16	0.12 ± 0.01	3.93	1.31 ± 0.13	0.12 ± 0.01	4.03	1.35 ± 0.17	0.12 ± 0.01	3.85
tCr	10.91 ± 0.66	–	1.62	10.89 ± 0.68	–	1.63	10.93 ± 0.65	–	1.61

GABA, Gamma-aminobutyric acid; Glu, Glutamate; Gln, Glutamine; Asp, Aspartate; GSH, Glutathione; Ins, Myo-inositol; Lac, Lactate; NAA, N-acetylaspartate; Scyllo, Scyllo-inositol; NAAG, N-acetylaspartylglutamate; tCh, Total choline (glycerophosphocholine and phosphocholine); tCr, Total creatine (creatine and phosphocreatine).

**Table 2:** Average and standard deviation of metabolite concentrations and ratios to total creatine across all participants, with separate data for males and females, alongside average CRLB values, presented for the insular voxel.

Insula	All sample, *N* = 160			Males, *N* = 65			Females, *N* = 95		
	Concentration (Mean ± SD)	Ratio to tCr (Mean ± SD)	Mean CRLB (%)	Concentration (Mean ± SD)	Ratio to tCr (Mean ± SD)	Mean CRLB (%)	Concentration (Mean ± SD)	Ratio to tCr (Mean ± SD)	Mean CRLB (%)
GABA	3.88 ± 0.6	0.32 ± 0.05	11.56	3.7 ± 0.54	0.3 ± 0.05	11.89	4 ± 0.61	0.33 ± 0.05	11.33
Glu	14.44 ± 1.34	1.18 ± 0.09	3.42	14.51 ± 1.48	1.17 ± 0.09	3.38	14.38 ± 1.24	1.18 ± 0.09	3.44
Gln	2.91 ± 0.65	0.24 ± 0.05	14.22	3.03 ± 0.63	0.24 ± 0.05	13.11	2.83 ± 0.65	0.23 ± 0.05	14.98
Asp	3.77 ± 0.44	0.31 ± 0.03	10.46	3.86 ± 0.47	0.31 ± 0.03	10.14	3.71 ± 0.41	0.31 ± 0.03	10.68
GSH	2.65 ± 0.23	0.22 ± 0.02	6.22	2.68 ± 0.24	0.22 ± 0.02	6.08	2.63 ± 0.22	0.22 ± 0.02	6.32
Ins	8.62 ± 0.96	0.7 ± 0.07	4.61	8.89 ± 0.95	0.72 ± 0.06	4.37	8.43 ± 0.93	0.69 ± 0.07	4.78
Lac	0.87 ± 0.32	0.07 ± 0.02	34.91	0.9 ± 0.33	0.07 ± 0.02	31.29	0.85 ± 0.31	0.07 ± 0.03	37.4
NAA	16.43 ± 1.05	1.34 ± 0.1	1.98	16.28 ± 1.12	1.32 ± 0.1	1.95	16.53 ± 0.99	1.36 ± 0.09	2
Scyllo	0.29 ± 0.11	0.02 ± 0.01	30.38	0.26 ± 0.1	0.02 ± 0.01	33.35	0.3 ± 0.12	0.03 ± 0.01	28.32
NAAG	1.46 ± 0.38	0.12 ± 0.03	18.91	1.49 ± 0.32	0.12 ± 0.03	12.98	1.44 ± 0.42	0.12 ± 0.04	23.04
tCh	2.76 ± 0.38	0.23 ± 0.03	2.98	2.8 ± 0.36	0.23 ± 0.03	2.92	2.73 ± 0.39	0.22 ± 0.03	3.01
tCr	12.26 ± 0.89	–	1.86	12.4 ± 1.06	–	1.8	12.16 ± 0.74	–	1.89

GABA, Gamma-aminobutyric acid; Glu, Glutamate; Gln, Glutamine; Asp, Aspartate; GSH, Glutathione; Ins, Myo-inositol; Lac, Lactate; NAA, N-acetylaspartate; Scyllo, Scyllo-inositol; NAAG, N-acetylaspartylglutamate; tCh, Total choline (glycerophosphocholine and phosphocholine); tCr, Total creatine (creatine and phosphocreatine).

### 2.4 Statistical analysis

Statistical analysis was performed using IBM SPSS Statistics 28.0.0.0 (190) and RStudio 2022.7.1.554. The normality, homoscedasticity and linearity of all variables were investigated using scatterplots. Correlations were computed by Spearman ‘s correlation coefficient and missing values were excluded in casewise fashion. Significance values are two-tailed and family-wise FDR corrected at α <0.05, unless stated otherwise. To investigate effects of biological sex on the main variables of interest Student’s two-sample t-test was used after testing the assumption of homogeneity of variances using Levene’s test. Test statistics and *p*-values are also supplemented by Bayes factors reported in standard form as the ratio of evidence for the alternative hypothesis and for the null hypothesis (BF10). A default noninformative effect size prior was used: a Cauchy distribution with a scale parameter of √2/2 ≈ 0.707.

To evaluate the relationship between responses on the aversive grating (VD-med) and neurotransmitter levels together with other psychological and biological variables, a backward stepwise regression, using the Bayesian Information Criterion (BIC; Schwarz, 1978), was conducted in several phases. In backward stepwise regression, a full model including all candidate predictor variables is first constructed, after which regressors are removed one by one based on whether a measure of relative model quality (in this case BIC) would be improved. Performing the model selection in phases allowed us to incorporate *a priori* assumptions into the process. For multiple linear regression, model coefficients are reported both in unstandardized (B) and standardized (beta) form to facilitate interpretation.

The first model included only the control low-frequency grating responses (VD-low) as a regressor to eliminate a broader underlying tendency to report sensory distortions and pre-cortical/ocular processes independent of local cortical excitability. Backward stepwise regression was then computed with the effects of biological sex (women coded as 1, men as -1) and neurotransmitter levels, as well as their two-way interactions, using the first model as a lower limit on the included model terms. A third model was then constructed with all other biological and psychological variables (CAPS and GSQ total scores, five NEO-FFI factor scores, eight MAIA-2 subscale scores, and hours slept before experiment), except those related to menstruation, using the second model as a lower limit on model terms.

After constructing the three models, a *post hoc* sensitivity analysis was conducted using G*Power version 3.1.9.7 (Faul et al., 2009) to determine the smallest increase in explained variance (R2) between the first and third models detectable with our sample size. Cohen’s f2 was used as a standardized measure of effect size. Additionally, we calculated the smallest possible R2 of the full model, given the achieved R2 of the control-only (first) model, using the formula for f2 as a local effect size (Selya et al., 2012).

Additionally, the role of menstrual cycle in visual distortions and comfort during the perception of aversive grating (VD-med and Comfort-med) was examined using a basic cosine regression model to ensure that the number of days since menstruation is treated as a cyclical variable (Pewsey et al., 2013).

## 3 Results

**Figure 3:**
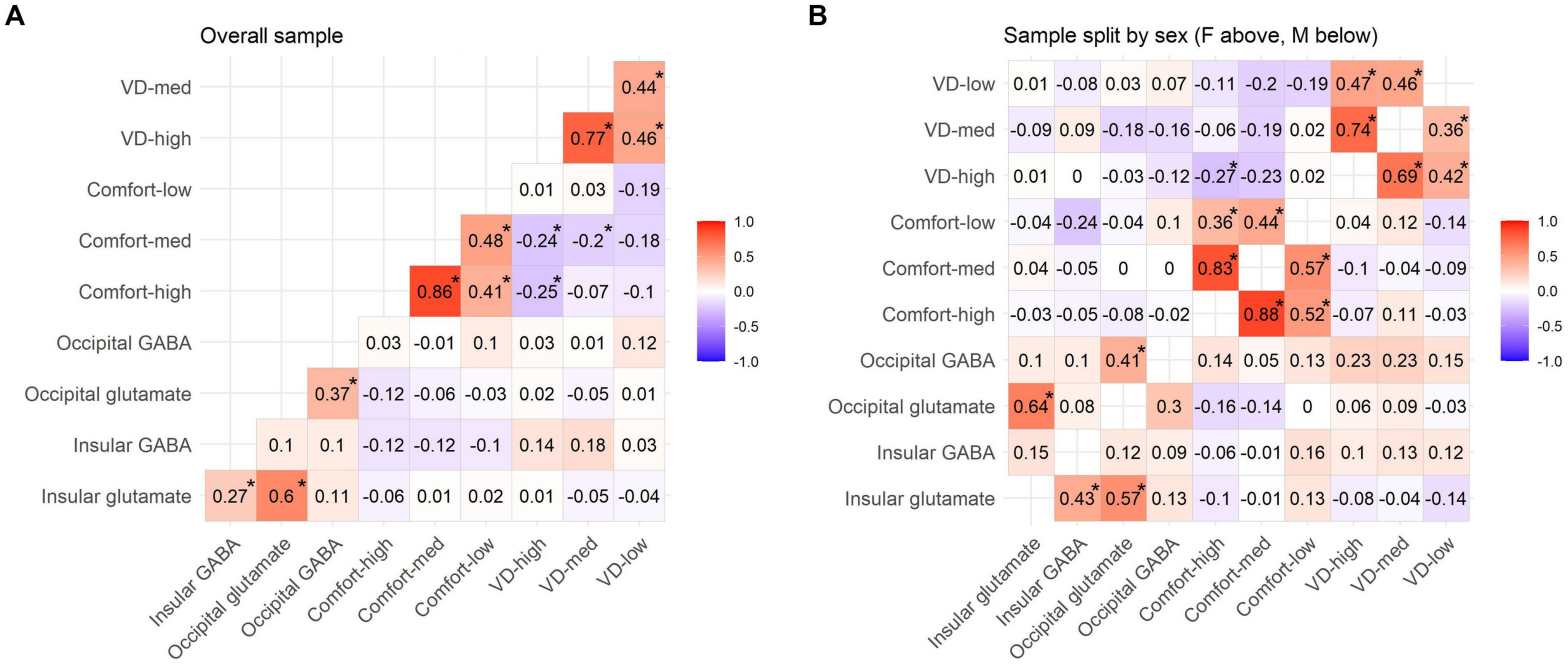
Pattern Glare Test scores and neurotransmitter levels (GABA/tCr and glutamate/tCr). Tables present non-parametric correlations for the **(A)** whole sample, **(B)** male (M) subsample and female (F) subsample. All the values marked with an asterisk are FDR corrected at *p* = 0.05.

The final sample included 160 young healthy volunteers (65 males, 95 females, age mean = 24.0, SD = 4.67). The main points of interest in spectroscopic analysis, GABA and glutamate, were correlated together positively in both occipital and insular voxel (Figure 3A). In the occipital voxel, the average absolute concentrations were 3.3 ± 0.55 [mM] for GABA, 10.1 ± 0.83 [mM] for glutamate, and relative concentrations GABA/total creatine (tCr; phosphocreatine plus creatine) were 0.3 ± 0.05 and glutamate/tCr were 0.93 ± 0.07; in the insular voxel, the average absolute concentrations were 3.89 ± 0.6 [mM] for GABA, 14.44 ± 1.34 [mM] for glutamate, and concentrations relative to total creatine were 0.32 ± 0.05 for GABA and 1.18 ± 0.09 for glutamate. The average signal-to-noise ratio was 70.26 ± 7.1 in V1 and 60.42 ± 5.4 in insula, and full width at half maximum (FWHM) was 0.03 ± 0.006 ppm (3.697 ± 0.739 Hz) for both voxels.

**Figure 4:**
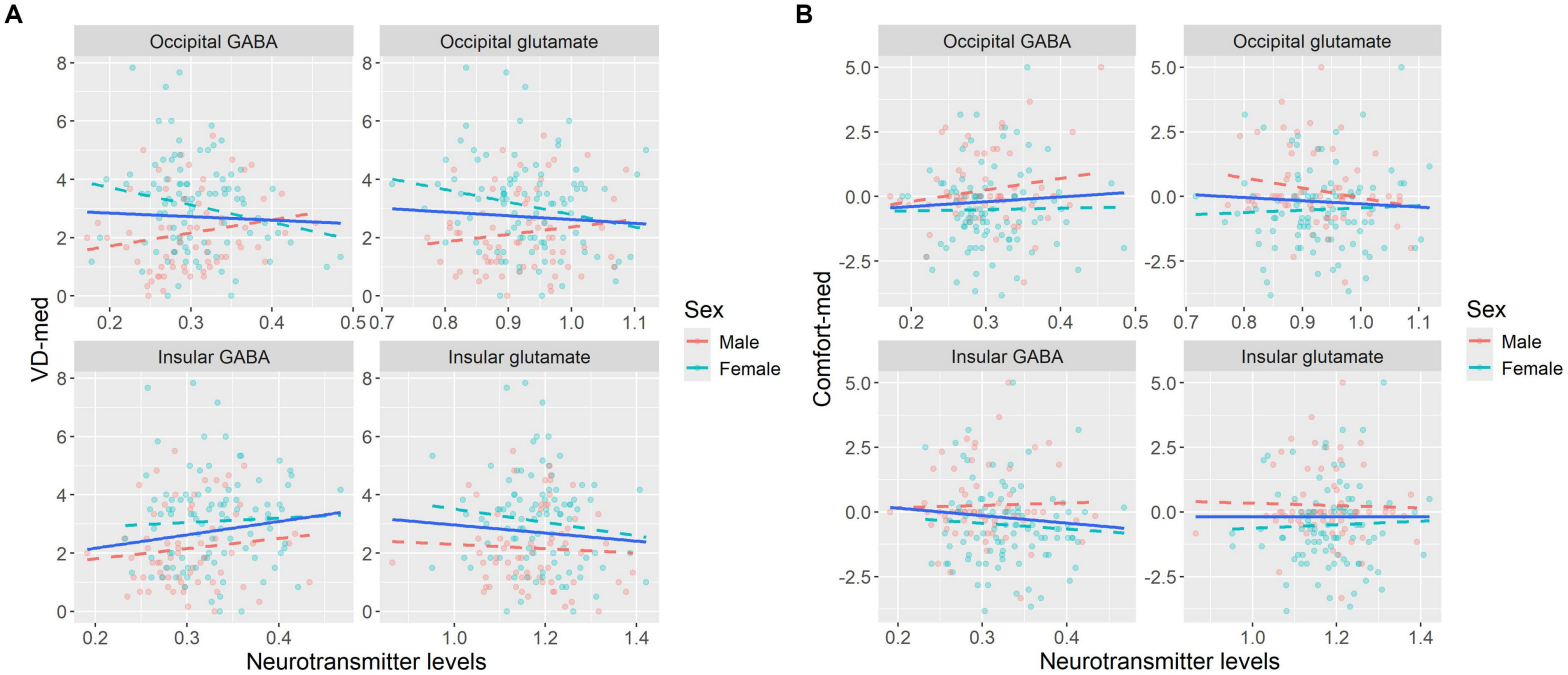
Graphic presentation of behavioral scores for aversive gratings plotted against neurotransmitter levels (GABA/tCr and glutamate/tCr), illustrating the trends for separate biological sexes for **(A)** VD-med and **(B)** Comfort-med. High visual sensitivity (visual distortions and discomfort) is hypothesized to be linked to high excitation (glutamate) and/or low inhibition (GABA). This expected pattern was not observed in either sex. Statistical values reflect Pearson’s correlation coefficient and 95% bootstrap confidence interval.

Overall, there was not a significant difference between the sexes in both occipital and insular glutamate/tCr levels, as well as occipital GABA/tCr (Student’s two-sample *t*-tests, all uncorrected *p* > 0.05 and BF10 < 0.33). However, men and women differed in insular GABA/tCr levels (*t* = −3.411, FDR corrected *p* < 0.01; BF10 = 32.566). Importantly, they also responded significantly differently to the key PGT variables, compared by the Student’s two-sample t-test (FDR corrected): VD-med (*t* = −3.795, *p* < 0.001; BF10 = 108.598), VD-high (*t* = −4.015, p < 0.001; BF10 = 228.127), Comfort-med (*t* = 3.131, *p* = 0.004) and Comfort-high (*t* = 2.247, *p* = 0.042). Descriptives for the PGT scores are presented in Table 3 and correlations between the PGT values in Figures 3A,B. The primary investigation focused on correlations between PGT scores and neurotransmitter levels in V1 and rAI (Figures 3, 4). Given the markedly different responses to the PGT scores in the two sexes, the general correlations are not truly relevant, and the correlations were calculated also for sample split by sex. Significant differences between the sexes can be seen in the correlations of PGT scores and occipital and insular neurotransmitter levels. Therefore, as the next step, regression models accounting for the effect of sex were constructed to reveal the true role of neurotransmitters on PGT scores, with sex considered as an independent variable.

**Table 3:** Behavioral PGT descriptives for the whole sample and separately for the two sexes.

	Mean	SD	Minimum	Maximum	Skewness	Kurtosis
All sample, *N* = 160						
VD-low	0.729	0.609	0.000	2.500	0.687	−0.221
VD-med	2.717	1.579	0.000	7.833	0.594	0.210
VD-high	2.608	1.439	0.000	7.167	0.725	0.232
Comfort-low	0.637	1.311	−1.667	5.000	1.523	1.667
Comfort-med	−0.196	1.553	−3.833	5.000	0.564	1.054
Comfort-high	−0.290	1.642	−4.500	5.000	0.498	1.003
Males, *N* = 65						
VD-low	0.589	0.507	0.000	2.000	0.821	0.086
VD-med	2.167	1.365	0.000	5.500	0.650	−0.507
VD-high	2.080	1.197	0.000	5.833	0.727	0.228
Comfort-low	0.653	1.253	−0.833	5.000	1.739	2.441
Comfort-med	0.256	1.426	−3.333	5.000	0.785	1.568
Comfort-high	0.058	1.558	−3.833	4.667	0.548	1.322
Females, *N* = 95						
VD-low	0.825	0.656	0.000	2.500	0.506	−0.530
VD-med	3.093	1.612	0.000	7.833	0.497	0.402
VD-high	2.968	1.484	0.333	7.167	0.625	0.001
Comfort-low	0.626	1.356	−1.667	5.000	1.429	1.383
Comfort-med	−0.505	1.567	−3.833	5.000	0.636	1.136
Comfort-high	−0.528	1.663	−4.500	5.000	0.567	1.098

**Figure 5:**
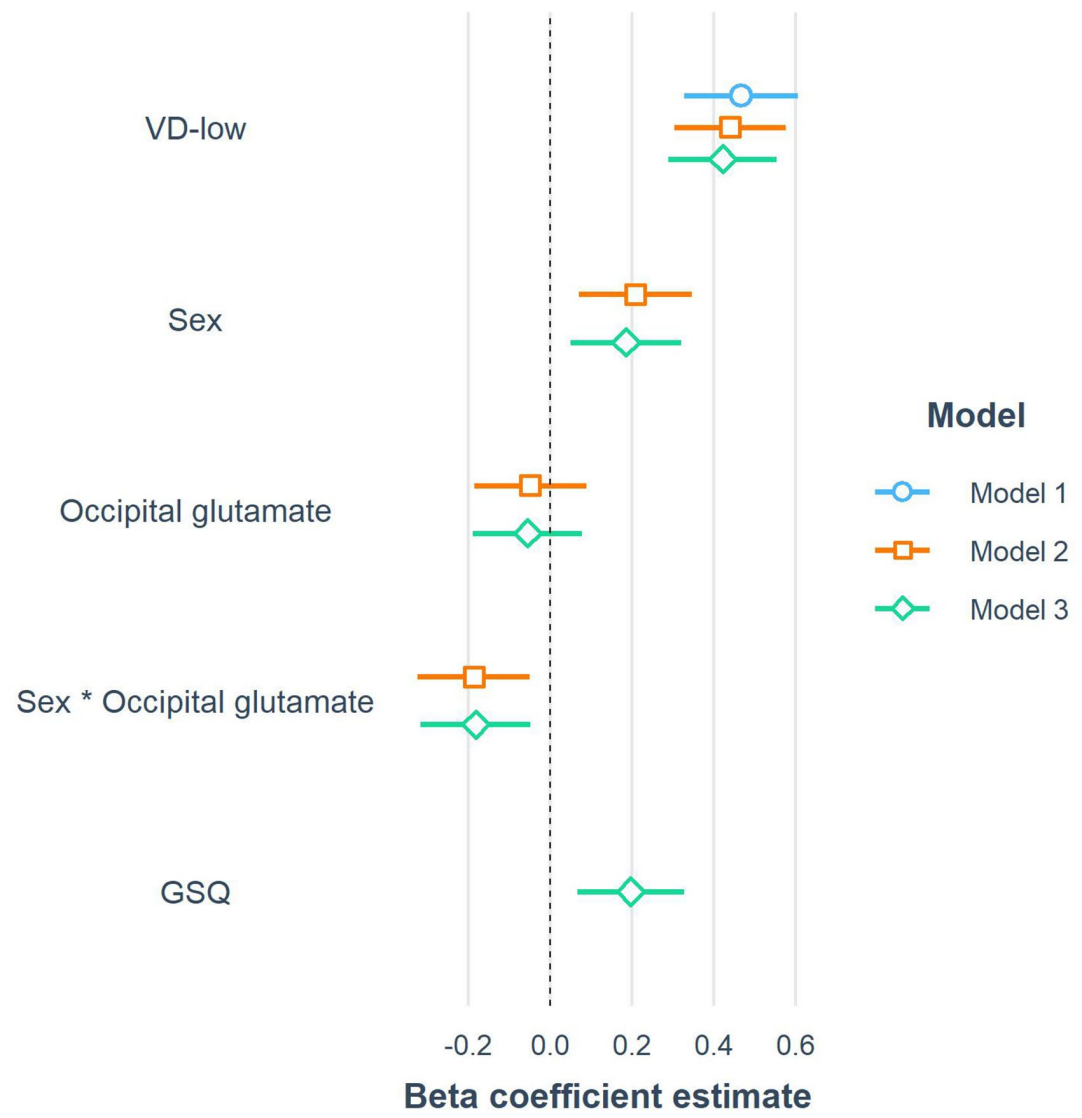
The coefficients of the three constructed linear models predicting the response to VD-med (medium-frequency gratings). Note that to improve readability, the coefficients in the forest plot were computed after the standardization of all continuous variables, including the response variable (beta coefficients). VD-low, low-frequency gratings; GSQ, Glasgow sensory questionnaire score.

The coefficients of the three constructed linear models investigating the predictors of visual distortions on VD-med are graphically presented in Figure 5. Firstly, VD-med was regressed on the control grating VD-low (Model 1). In the second phase (Model 2), the model assessing the relationship with neurotransmitters selected only the occipital glutamate and its interaction with biological sex as a predictor contributing to the overall score. From the biological and psychological variables, only the GSQ overall score s remained in the final model (Model 3).

Outcomes of the third final model show that in the overall sample, there was a non-significant negative relationship between occipital glutamate and VD-med, with an increase of 0.1 of Glu/tCr corresponding to -0.15 less distortions on average (B = −1.149; beta = −0.054). However, there was a significant (*p* < 0.01) interaction between occipital glutamate and sex, with women having a more negative relationship between glutamate and VD-med than men (B = −3.84; beta = −0.181), while they generally reported more visual distortions on the aversive VD-med grating than men (B = 3.852; beta = 0.186). As the only included biological or psychological variable, GSQ scores had a statistically significant (*p* < 0.01) positive association with VD-med (B = 0.021; beta = 0.198). The first model (control-only) explained 21.84% of the variance in VD-med scores. The second model, which included occipital glutamate and its interaction with sex, accounted for 30.06% of the variance. The third model, which added GSQ, explained 33.87% of the variance in VD-med scores.

A *post hoc* sensitivity analysis was conducted to assess the difference in explained variance between Model 1 and Model 3 using the G*Power software. The analysis assumed an alpha level of 0.05, a power of 0.8, and a sample size of 160. The full model included five predictors, compared against a control-only model with one predictor. Under these assumptions, effect sizes larger than f2 = 0.077 can be reliably detected, which falls between Cohen’s (1988) criteria for a small effect size (0.02) and a medium effect size (0.15). Given that Model 1 explains 21.84% of the variance in the dependent variable VD-med, this effect size corresponds to the full model needing to explain R2 = 27.42% or more.

**Figure 6:**
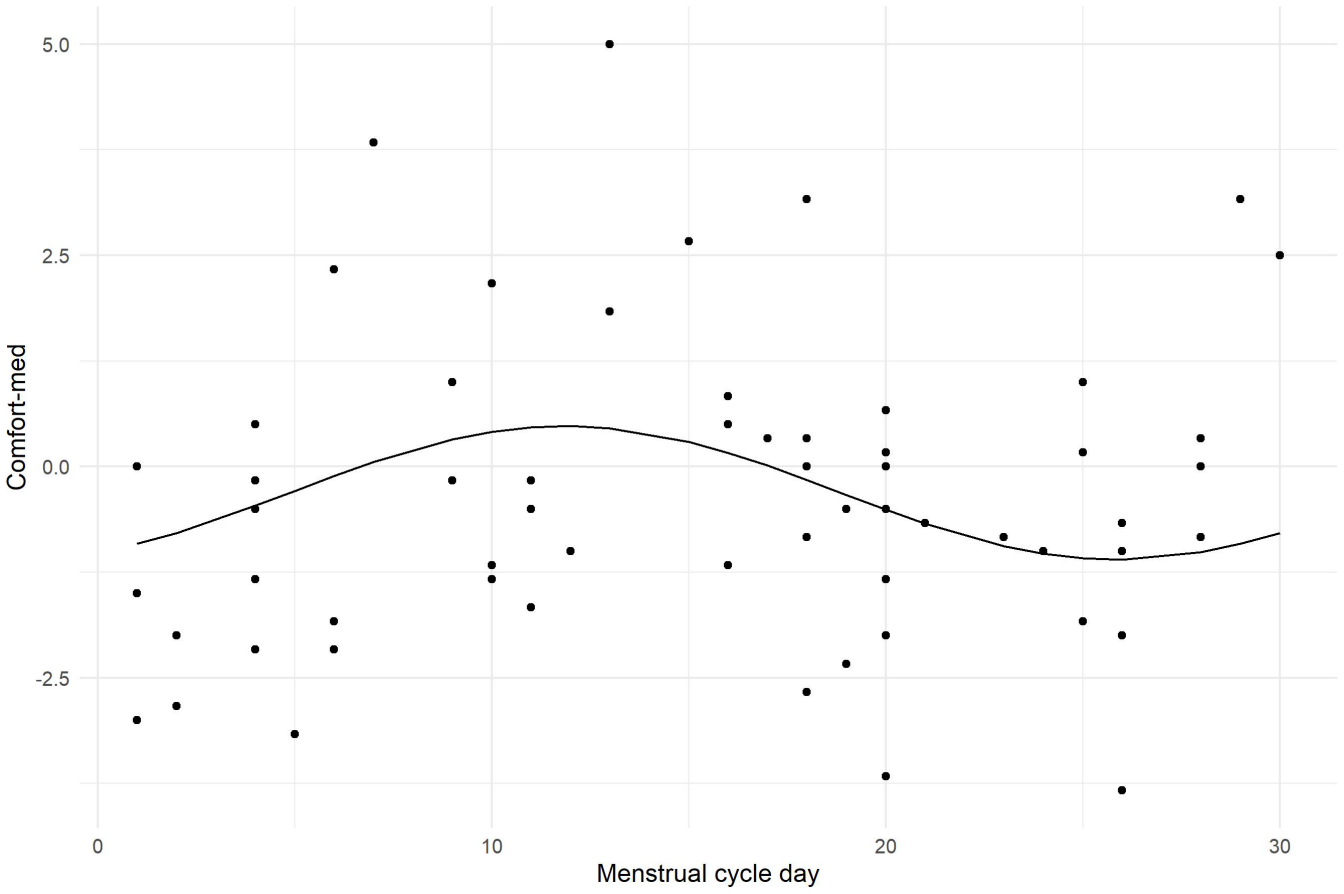
Plot illustrating the outcome of the cosine regression model evaluating the effect of the menstrual cycle on Comfort-med.

Next, the cosine regression model evaluated the effect of the menstrual cycle on scores of aversive grating. Since the variables concerning menstruation were available only for a part of the sample, they could not be included in multivariate linear models. Their effects were therefore examined separately in a relevant subsample. Sixty-eight women reported having a normal menstrual cycle, while twenty-seven did not. There was no statistically significant difference (at α = 0.05) between these groups in any of the PGT scores or neurotransmitter levels. Scores in VD-med were not predicted by the menstrual cycle (F_2,58_ = 0.964; *p* = 0.388). However, the cosine model of a cyclical relationship between the day of menstruation cycle and Comfort-med seemed to capture a non-significant trend (F_2,58_ = 2.728; *p* = 0.074), explaining 8.6% of the variance in comfort ratings. The model is visualized using a scatterplot and a regression curve in Figure 6.

## 4 Discussion

### 4.1 Factors affecting perception of aversive spatial frequency

Despite being well-powered, our study did not confirm the hypothesis of a straightforward relationship between V1 occipital or insular neurotransmitter levels and the Pattern Glare Test as a selected proxy measure of visual sensitivity in neurotypical adults. However, a highly significant pattern of biological sex moderating this association emerged in our dataset. To our knowledge, this is the first work on cortical excitability and visual sensitivity to describe such an interaction. The differences between sexes’ responses on the PGT were addressed and taken into consideration in further examination by correlations and linear regression modeling. Although both GABA and glutamate in the primary visual cortex were weakly negatively correlated with visual distortions on aversive medium-frequency grating of 3 cpd in women, this correlation had a positive trend in men. When controlled for sex in the regression modeling, GABA was not included in the final model. On the contrary, our model revealed the predictive power of occipital glutamate, but only when an interaction with biological sex was modeled. This suggests that its role is more important in visual sensitivity than GABA. The role of insular neurotransmitters in the perception of aversive gratings was not supported by the model’s outcomes.

As far as responses in the primary visual cortex are concerned, our findings do not uphold the assumption of a direct involvement of GABA and glutamate levels in subjective visual sensitivity, suggesting the hyperexcitability hypothesis requires refinement. Furthermore, although relatively weak, the direction of the relationship was opposite to what would be expected, both on the sample level and in the female subgroup, with larger resting glutamate levels in V1 corresponding to the experience of *fewer* visual distortions. These paradoxical findings might result partly from the neuroimaging method used. Magnetic resonance spectroscopy provides information on baseline neurochemical levels in subjects’ neuronal cytoplasm, but does not quantify synaptic neurotransmitter activity (Stagg et al., 2011b; Duncan et al., 2014), which is more likely than total metabolite concentrations to be directly related to perceptual responses (Chan et al., 2022). In previous research that we built upon, significant results were achieved only after modulating basic cortical excitability through neurostimulation methods such as transcranial direct current stimulation (tDCS), or directly during the PGT. Our findings suggest that task-related visual sensitivity in neurotypical adults may be influenced by underlying cortical processes beyond simple quantification of neurotransmitter concentrations during resting state—individuals prone to pattern glare could show signs of elevated cortical excitability only after being exposed to aversive patterns and their baseline neurotransmitter levels measured in a separate MRS session do not play a critical role in their subjective PGT scores.

Another possible explanation lies within the examined test subjects. The imbalance between excitatory and inhibitory mechanisms in relation to sensory sensitivity was described in studies of wide range of neurological and neurodevelopmental disorders including migraine (Aurora and Wilkinson, 2007; Nguyen et al., 2016), epilepsy (Wilkins et al., 2004), autism spectrum disorder (Dickinson et al., 2016; Wood et al., 2021), depression (Qi et al., 2019; Wang et al., 2022), or anxiety (Hui et al., 2023). However, the expected relationship between the Pattern Glare Test and neurotransmitter levels in V1 of the visual cortex may not be sufficiently robust in the neurotypical individuals to reliably deduce GABA or glutamate as a reliable indicator of visual discomfort. Additional factor possibly affecting the outcomes could be that our study sample generally scored low on the susceptibility to aberrant experiences, as shown by the CAPS questionnaire (mean = 6.32; SD = 4.95).

Apart from neurotransmitters, the modulatory role of only a single psychological variable was revealed: trait-based sensory sensitivity (GSQ). Other variables, including susceptibility to anomalous perceptions (CAPS), perception of body sensations (MAIA-2), personality factors (NEO-FFI), and sleep, were not included as relevant by the constructed models.

In women, the day of the menstrual cycle affected the comfort rating; the closer to ovulation, the higher the comfort, which then gradually decreased during the luteal phase and was the lowest at the beginning of the menstrual phase. This is in accordance with the progesterone-derived neurosteroids inhibitory effect during the follicular menstrual phase caused by the increase in the GABAergic inhibition (Smith et al., 2002), decrease in glutamate excitation and inhibition of pyramidal neurons (Stahl, 2008), which can possibly reduce the feeling of subjective discomfort while observing the aversive patterns.

### 4.2 Sex differences in subjectively reported visual stress

Our findings make a novel and noteworthy contribution to examining individual predisposition to pattern glare effects of visual discomfort. However, the complexity of the relationship between neurotransmitters and reported visual stress by the two sexes is challenging. There was no statistical difference between the sexes in occipital neurotransmitter levels, the difference in means was only found for the PGT variables. We found no pattern in psychological traits examined in this work that explains these differences. A comprehensive investigation of the Pattern Glare Test carried out by Evans and Stevenson (2008) with the objective of establishing standard testing norms indicated that while pattern glare correlates with conditions such as migraines, which exhibit a higher prevalence in women, their study did not identify substantial gender disparities in behavioral responses. However, it is worth mentioning that their sample comprised 33 females and 33 males with notably broad age ranges in both groups (48 ± 21 years; range: 12–82 years; 48 ± 25 years; range: 10–90 years, respectively), which differ substantially from those in our study and also included children. The same study revealed that the effect of PGT decreases with age significantly at both medium and high-frequency patterns. This leads us to speculate that sex differences might have been present in young adults in the age range used in our study but were statistically mitigated by age effects. Although there are a few studies that considered the potential influence of biological sex on the PGT scores in their study design by gender-matching the sample (e.g., Allen et al., 2010; Beasley and Davies, 2012; Qi et al., 2019), no study known to us that utilized Pattern Glare Test as a proxy measure of visual stress considered sex as a possible covariate during the analysis. Yet, an emerging number of recent studies propose the importance of control for sex in vision research (Shaqiri et al., 2018), whether the arguments arises from addressed differences in perception of color (Johansson et al., 2018; Abramov et al., 2012a; Fider and Komarova, 2019), visual acuity (Abramov et al., 2012b), contrast sensitivity (Foutch and Peck, 2013), or motion perception (Ruggeri et al., 2020). Considered together with the sex-contradictory results of this study, involvement of both sexes equally and inclusion of sex as a factor in the statistical analyses of future PGT studies could bring new insight into this area.

The present study has a few methodological limitations. First, the sex differences observed in subjective responses played a significant role in disentangling the actual role of neurotransmitters, thus the behavioral responses could not be easily explained by correlations. Although the study was performed on a very large sample, further research should be performed to replicate these results in a different neurotypical sample, given that previous studies did not identify the observed inter-sex differences in PGT scores. In addition, it would be useful to conduct a study on neurodiverse or neurological clinical samples that have been previously investigated in visual sensitivity research, as this could improve our understanding of the factors influencing the results of this study. The impact of sex differences should be considered in the study design, while controlling for the biological variables, such as menstrual cycle. Second, although this study was focused on the relationship between the pattern glare scores and the neurotransmitter levels, concentrations were not obtained directly during the visual task. There is evidence for differences in these levels during the different conditions, e.g., GABA decreases whereas Glx (glutamate + glutamine) levels increase with increasing visual input (Kurcyus et al., 2018). Our results showed that decreased glutamate levels correlate with increased number of visual distortions, but this could be claimed only for its resting state with closed eyes. It would be useful to verify this relationship with spectroscopy measurement during the PGT. Moreover, based on previous literature, we believe that the SPECIAL sequence is capable of providing reliable GABA measurements. However, it would be useful for future work to validate the current findings using more conventional GABA measurement techniques (i.e., MEGA-PRESS). Lastly, we suggest expanding the scope of investigation within the visual cortex to encompass the association cortex. Previous research on visual discomfort among migraine patients has indicated a notable decrease in cortical activation within areas V2-V4 when utilizing colored lenses, contrasting with findings in V1 (Huang et al., 2011). This implies that exploring the hyperexcitability of association visual cortex in neurotypical subjects could provide fresh insights into the underlying neural mechanisms influencing heightened reactions to aversive visual stimuli, as the visual association cortex may mediate such effects more than V1.

## Data Availability

The R script for statistical analysis is available in the Zenodo repository at: 10.5281/zenodo.12208682.
